# O Impacto da Aptidão Cardiorrespiratória no Paradoxo da Obesidade em Insuficiência Cardíaca com Fração de Ejeção Reduzida

**DOI:** 10.36660/abc.20190337

**Published:** 2020-10-13

**Authors:** Rita Ilhão Moreira, Tiago Pereira Silva, António Valentim Gonçalves, Joana Feliciano, Pedro Rio, Rui Soares, Rui Cruz Ferreira

**Affiliations:** 1 Hospital de Santa Marta Lisboa Portugal Hospital de Santa Marta, Lisboa – Portugal

**Keywords:** Insuficiência Cardíaca, Obesidade, Índice de Massa Corporal, Exercícios Respiratórios, Fração de Ejeção Ventricular, Aptidão Cardiorrespiratória, Testes de Função Respiratória

## Abstract

**Fundamento:**

Índice de massa corporal (IMC) elevado tem sido associado a desfechos melhores em pacientes com insuficiência cardíaca com fração de ejeção reduzida. Este achado tem levado ao conceito do paradoxo da obesidade.

**Objetivo:**

Investigar o impacto de tolerância ao exercício e capacidade cardiorrespiratória no paradoxo da obesidade.

**Método:**

Pacientes ambulatoriais com insuficiência cardíaca sintomática e fração de ejeção ventricular esquerda (FEVE) ≤ 40%, acompanhados no nosso centro, foram prospectivamente submetidos à avaliação abrangente de linha de base incluindo parâmetros clínicos, laboratoriais, eletrocardiográficos, ecocardiográficos e de exercício cardiopulmonar. A população do estudo foi dividida de acordo com o IMC (< 25, 25 – 29,9 e ≥ 30 kg/m^2^). Todos os pacientes foram acompanhados durante 60 meses. O desfecho composto foi definido como morte cardíaca, transplante cardíaco urgente ou necessidade de suporte circulatório mecânico. Valores de p < 0,05 foram considerados significativos.

**Resultados:**

Dos 282 pacientes incluídos (75% masculino, 54 ± 12 anos, IMC 27 ± 4 kg/m^2^, FEVE 27% ± 7%), o desfecho composto ocorreu em 24,4% durante o acompanhamento. Os pacientes com IMC elevado eram mais velhos e apresentavam FEVE e níveis séricos de sódio mais elevados, bem como menor inclinação de eficiência ventilatória (VE/VCO_2_). VE/VCO_2_ e consumo de oxigênio de pico (VO_2_p) eram fortes preditores prognósticos (p < 0,001). Na análise univariada de regressão de Cox, o IMC elevado foi associado a desfechos melhores (razão de risco 0,940, intervalo de confiança 0,886 – 0,998, p 0,042). Porém, após ajustar para ou inclinação VE/VCO_2_ ou VO_2_p, o papel protetor do IMC sumiu. O benefício de sobrevida do IMC não foi evidente quando os pacientes foram agrupados de acordo com a classe de aptidão cardiorrespiratória (VE/VCO_2_, valor de corte de 35, e VO_2_p, valor de corte de 14 mL/kg/min).

**Conclusão:**

Estes resultados sugerem que a aptidão cardiorrespiratória supera a relação entre o IMC e a sobrevida em pacientes com insuficiência cardíaca.

## Introdução

A obesidade impacta a maioria dos fatores de risco para doenças cardiovasculares e é um fator de risco independente para o desenvolvimento de insuficiência cardíaca (IC), estando presente em aproximadamente 20% a 30% dos pacientes com IC avançada.^1^^–^^3^ Apesar disso, vários investigadores têm demonstrado que o índice de massa corporal (IMC) elevado está paradoxalmente associado a melhores resultados clínicos no contexto de IC estabelecida, fenômeno que foi denominado “paradoxo da obesidade.^4^^–^^6^

Diversos mecanismos concorrentes e frequentemente contraditórios foram propostos para explicar o paradoxo da obesidade na IC. Os possíveis motivos incluem níveis elevados de lipoproteínas séricas,^7^ níveis baixos de adiponectina^8^ e resposta diminuída à ativação simpática.^9^ Fatores de confusão também têm sido sugeridos como uma explicação potencial.^10^

A aptidão cardiorrespiratória, medida de várias maneiras como consumo de oxigênio de pico (VO_2_p) ou inclinação de eficiência ventilatória (inclinação VE/VCO_2_), tem sido identificada como um importante preditor de sobrevida na IC.^11^^,^^12^ Um forte paradoxo da obesidade tem sido demonstrado em pacientes com doença cardíaca coronária,^13^^,^^14^ mas não em pacientes com altos níveis de tolerância ao exercício.^15^^,^^16^

Nós visamos investigar o impacto de tolerância ao exercício e capacidade cardiorrespiratória no paradoxo da obesidade.

## Métodos

A investigação está de acordo com os princípios prescritos na Declaração de Helsinque. Todos os participantes preencheram um termo de consentimento livre e esclarecido e o comitê de ética institucional aprovou o protocolo do estudo.

### Seleção de pacientes e avaliação complementar

Realizamos um estudo de coorte prospectivo incluindo todos os pacientes com IC com fração de ejeção reduzida (ICFEr) (≤ 40%), em classe II ou III da New York Heart Association (NYHA), acompanhados nas Clínicas de Insuficiência Cardíaca da nossa instituição. Todos os pacientes encaminhados à Clínica de Insuficiência Cardíaca foram submetidos à abrangente avaliação complementar, de 2000 a 2009. Foram coletados prospectivamente os dados clínicos, laboratoriais, eletrocardiográficos, ecocardiográficos e de exercício cardiopulmonar; todos os exames foram realizados no prazo de um mês para cada paciente. Foram excluídos os pacientes com os seguintes fatores: menores de 18 anos, revascularização coronária percutânea ou cirurgia cardíaca programada, comorbidades limitadoras de exercício (incluindo doença cerebrovascular, comprometimento musculoesquelético e doença vascular periférica grave) e transplante cardíaco prévio.

Foi realizado o teste de exercício cardiopulmonar máximo em esteira limitado por sintomas usando o protocolo de Bruce modificado (esteira GE Marquette Series 2000). Ventilação por minuto, consumo de oxigênio e produção de dióxido de carbono foram aferidos respiração a respiração, usando um analisador de gases SensorMedics Vmax 229. Antes de cada teste, o equipamento foi calibrado de forma padrão utilizando gases de referência. Os pacientes foram incentivados a realizar exercício até que a relação de troca respiratória (relação entre a produção de dióxido de carbono e o consumo de oxigênio, RER) fosse ≥ 1,10. Foi definido o VO_2_p como a maior média de 30 segundos alcançada durante o exercício e foi normalizado para a massa corporal; substituto para massa magra foi considerado em pacientes obesos (IMC ≥ 30 kg/m^2^). A porcentagem de VO_2_p previsto foi calculada de acordo com Hansen et al.,^17^ Foi calculada a inclinação VE/VCO_2_ por regressão linear de mínimos quadrados, utilizando dados obtidos ao longo de todo o exercício.^18^ Os dados eletrocardiográficos foram interpretados por um médico durante o exame. Foram obtidos o peso e a estatura em uma balança antropométrica Welmy 110-CH, antes de realizar o teste cardiopulmonar.

Foi utilizado um sistema de ultrassom GE Vivid 9 para obter vistas paraesternais nos eixos longo e curto, bem como vistas apicais de duas, três e quatro câmaras. Os parâmetros ecocardiográficos, incluindo os volumes diastólicos e sistólicos finais do ventrículo esquerdo e a fração de ejeção do ventrículo esquerdo, foram determinados de acordo com as recomendações da Sociedade Americana de Ecocardiografia.

### Acompanhamento e desfecho

Todos os pacientes foram acompanhados durante 60 meses. Os pacientes foram avaliados quanto a ocorrência de óbito, transplante cardíaco ou necessidade de suporte circulatório mecânico. Os dados foram obtidos de consultas ambulatoriais e revisão dos prontuários médicos, com entrevista telefônica padronizada complementar para todos os pacientes aos 12, 36 e 60 meses de acompanhamento.

O desfecho composto foi definido como morte cardíaca, transplante cardíaco urgente (ocorrendo durante internação não planejada por agravamento da CI, requerendo inotrópicos) ou necessidade de suporte circulatório mecânico.

### Análise estatística

Os pacientes foram divididos nos seguintes três grupos de acordo com o IMC: < 25, 25 – 29,9 e ≥ 30 kg/m^2^. A aptidão cardiorrespiratória foi dicotomizada em baixo e alto risco de acordo com VE/VCO_2_ (valor de corte de 35^19^) e VO_2_p (valor de corte de 14 mL/kg/min^11^).

Os dados categóricos são apresentados como frequências (porcentagens) e as variáveis contínuas como média (desvio padrão), conforme apropriado. As variáveis contínuas foram analisadas usando o teste t de Student não pareado após verificação da normalidade (teste de Kolmogorov-Smirnov). Foram analisadas as variáveis categóricas usando o teste quiquadrado ou o teste exato de Fisher. A análise de variância (ANOVA) unilateral foi usada para comparação entre grupos, quando apropriado. Foram aplicados modelos univariados e multivariado de regressão de Cox para analisar o tempo até o desfecho composto. A sobrevida foi estimada pela análise de Kaplan-Meier e comparada pelo teste de log-rank. Foi realizada análise adicional do grupo de IMC menor (< 25 kg/m^2^), separando IMC < 20 e IMC 20 – 24,9 kg/m^2^. Porém, devido à pequena porcentagem de pacientes com IMC < 20 kg/m^2^ (apenas 17 pacientes), apenas as características de linha de base foram avaliadas (Tabela Suplementar S1 e Figura Suplementar S1) e nenhuma análise estatística adicional foi realizada. Todos os testes estatísticos foram bilaterais. Foi considerado significativo o valor de p < 0,05. O software SPSS versão 21 (SPSS Inc., Chicago, Illinois, EUA) foi usado para a computação.

## Resultados

Foram incluídos 282 pacientes, com idade média de 53,7 ± 12,1 anos; 75,5% eram de sexo masculino, com IMC médio de 26,8 ± 4,3 kg/m^2^, e 37,6% tinham cardiomiopatia isquêmica. A fração de ejeção ventricular esquerda (FEVE) média foi de 27,4% ± 7,3% e 23,0% de pacientes estavam em classe ≥ III NYHA. Em relação à terapia, 96,8% dos pacientes estavam sob uso de um inibidor da enzima de conversão da angiotensina ou um bloqueador do receptor da angiotensina; 80,1% estavam sob uso de um betabloqueador; 68,1% estavam recebendo um antagonista mineralocorticoide e 26,2% tinham estimulação biventricular. Todos os pacientes foram acompanhados durante 60 meses. O desfecho composto de morte cardíaca, transplante cardíaco urgente ou necessidade de suporte circulatório mecânico ocorreu em 24,4% dos pacientes.

### Grupos de índice de massa corporal

São apresentadas na Tabela 1 as características de linha de base dos pacientes de acordo com os grupos de IMC. Os pacientes com IMC mais alto eram mais velhos e apresentavam FEVE e níveis séricos de sódio mais elevados. O esforço do exercício foi, em média, máximo em todos os grupos de IMC (RER > 1.05), embora o IMC mais alto estivesse associado a um valor menor de RER. O IMC elevado foi associado a menor inclinação VE/VCO_2_ (p 0,005), bem como VO_2_p numericamente mais alto e percentual do VO2p previsto, embora sem alcançar significância estatística.

**Tabela 1:** Características de linha de base de acordo com a classe de IMC

Características de linha de base	IMC < 25 kg/m^2^ (n = 99)	IMC 25 – 29,9 kg/m^2^ (n = 119)	IMC ≥ 30 kg/m^2^ (n = 64)	p
Idade, anos, média (DP)	49,0 (± 9,6)	59,3 (± 4,5)	57,3 (± 8,5)	0,022
Sexo masculino, n (%)	70 (70,7%)	92 (77,3%)	51 (79,7%)	0,359
Etiologia isquêmica, n (%)	37 (37,4%)	44 (37,0%)	25 (39,1%)	0,961
Diabetes mellitus, n (%)	8 (8,0%)	30 (25,2%)	22 (35,1%)	<0,001
FEVE, % média (DP)	24,0 (± 5,2)	28,3 (± 0,6)	27,8 (3,6)	0,003
Classe ≥ III NYHA, n (%)	29 (29,6%)	24 (20,3%)	12 (19,0%)	0,184
IECA ou BRA, n (%)	98 (99,0%)	113 (95,0%)	62 (96,9%)	0,241
Betabloqueadores, n (%)	75 (75,8%)	99 (83,2%)	53 (82,8%)	0,335
Antagonista mineralocorticoide, n (%)	63 (63,6%)	86 (72,3%)	44 (68,6%)	0,364
Estimulação biventricular, n (%)	21 (21,2%)	33 (27,7%)	19 (29,7%)	0,402
DCI, n (%)	23 (23,2%)	30 (25,2%)	16 (35,0%	0,938
Hb, g/dL, média (DP)	15,0 (± 1,3)	12,4 (± 1,1)	13,6 (± 1,7)	0,075
TFGe, mL/min/1,73 m^2^, média (DP)	103,4 (± 48,5)	69,0 (± 23,3)	73,0 (±23,5)	0,140
Sódio, mEq/L, média (DP)	134,5 (± 7,1)	139,0 (±2,6)	136,4 (± 4,8)	0,025
BNP, pg/mL, média (DP)	534,3 (± 365,3)	350,7 (± 89,0)	573,4 (± 300,6)	0,710
RER, média (DP)	1,13 (± 0,14)	1,06 (± 0,49)	1,07 (± 0,15)	0,023
VO_2_p, mL/kg/min, média (DP)	15,0 (± 2,6)	15,2 (± 3,9)	16,1 (± 2,8)	0,758
% VO_2_p previsto, % média (DP)	43,0 (± 8,4)	55,3 (± 9,3)	60,3 (± 16,1)	0,207
Inclinação VE/VCO_2_, média (DP)	43,4 (± 6,6)	33,8 (± 6,0)	33,1 (± 8,1)	0,005

BNP: peptídeo natriurético cerebral; BRA: bloqueador do receptor da angiotensina; DCI: desfibrilador cardioversor implantável; FEVE: fração de ejeção ventricular esquerda; Hb: hemoglobina; IECA: inibidor da enzima de conversão da angiotensina; IMC: índice de massa corporal; inclinação VE/VCO_2_: inclinação de eficiência ventilatória; NYHA: New York Heart Association; RER: relação de troca respiratória; TFGe: taxa de filtração glomerular estimada; VO_2_p: consumo de oxigênio de pico. P calculado por análise de variância.

**Figura 1:**
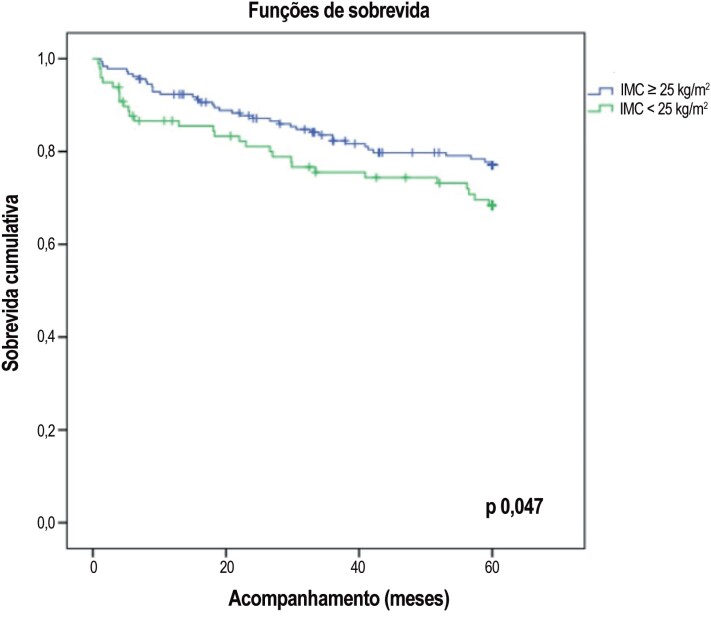
Análise Kaplan-Meier de acordo com índice de massa corporal (IMC) no grupo geral.

Em um modelo não ajustado de riscos proporcionais de Cox, o IMC foi um preditor de sobrevida livre de eventos quando expresso como uma variável contínua (razão de risco [RR] 0,940, IC 0,886 – 0,998, p 0,042, Tabela 2) ou uma variável dicotômica (log-rank valor de p 0,047, Figura 1).

**Tabela 2:** Desfecho composto de acordo com índice de massa corporal e parâmetros de teste de exercício cardiopulmonar

Variável dependente	RR (95% IC)	p
IMC, não ajustado	0,940 (0,886 – 0,998)	0,042
Inclinação VE/VCO_2_, não ajustada	1,164 (1,135 – 1,194)	< 0,001
VO_2_p, não ajustado	0,791 (0,742 – 0,842)	< 0,001

IMC: índice de massa corporal; inclinação VE/VCO2: inclinação de eficiência ventilatória; RR: razão de risco; VO_2_p: consumo de oxigênio de pico.

### Aptidão cardiorrespiratória

Tanto a inclinação VE/VCO_2_ quanto o VO_2_p foram preditores fortes de sobrevida livre de eventos na análise univariada (p < 0,001, Tabela 2).

**Figura 2:**
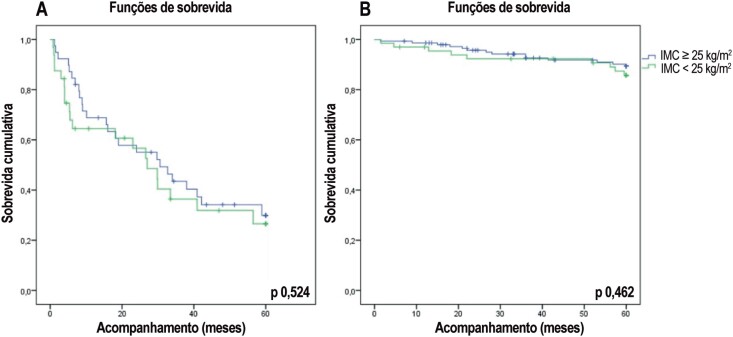
Análise Kaplan-Meier de acordo com índice de massa corporal (IMC) nos grupos (A) de baixa aptidão cardiorrespiratória (inclinação VE/VCO_2_ > 35) e (B) de alta aptidão cardiorrespiratória (inclinação VE/VCO_2_ ≤ 35).

Quando os pacientes foram agrupados em classes de aptidão cardiorrespiratória de baixo e alto risco de acordo com a inclinação VE/VCO_2_, o IMC não foi um preditor de desfechos clínicos na análise univariada de regressão de Cox (p 0,771 para inclinação VE/VCO_2_ > 35 e p 0,439 para inclinação VE/VCO_2_ ≤ 35). A Figura 2 ilustra as características de sobrevida livre de eventos de cada grupo de aptidão cardiorrespiratória. Além disso, o IMC não afetou sobrevida livre de eventos quando os pacientes foram agrupados por VO_2_p (p 0,170 para VO_2_p ≤ 14 mL/kg/min e p 0,164 para VO2p > 14 mL/kg/min).

Embora o IMC tenha sido um preditor prognóstico na análise univariada, após usar uma análise de regressão de Cox ajustando para a inclinação VE/VCO_2_, o IMC perdeu a sua capacidade prognóstica (p 0,786, Tabela 3). Além disso, não houve relação entre o IMC e a sobrevida livre de eventos após ajustar para VO_2_p (p 0,201, Tabela 3).

**Tabela 3:** Desfecho composto de acordo com índice de massa corporal ajustado para parâmetros de teste de exercício cardiopulmonar

Variável dependente	RR (95% CI)	p
IMC, ajustado por VE/VCO_2_	1,008 (0,949 – 1,072)	0,786
IMC, ajustado por VO_2_p,	0,949 (0,892 – 1,020)	0,201

IMC: índice de massa corporal; inclinação VE/VCO2: inclinação de eficiência ventilatória; RR: razão de risco; VO_2_p: consumo de oxigênio de pico.

## Discussão

Neste estudo, avaliamos se a capacidade cardiorrespiratória afeta o paradoxo da obesidade. Os achados podem ser resumidos da maneira seguinte: (1) o paradoxo da obesidade está presente nesta população com IC; (2) a inclinação VE/VCO_2_ e o VO_2_p são fortes preditores prognósticos, e (3) mais importante, a capacidade prognóstica do IMC se perde ao se considerar qualquer um desses dois parâmetros de aptidão cardiorrespiratória.

A obesidade é um dos principais fatores de risco para o desenvolvimento de IC. No Estudo Framingham Heart, com 5.881 participantes, o risco de IC dobrou em indivíduos obesos (RR 1,90 para homens e RR 2,12 para mulheres).^20^ Estes resultados foram semelhantes em estudos maiores, incluindo um estudo com mais de 59.000 participantes livres de IC no início, onde os RRs ajustados multivariados para desenvolver IC com diferentes níveis de IMC (< 25, 25 – 29,9 e ≥ 30 kg/m^2^) foram de 1,00, 1,25 e 1,99 para homens e 1,00, 1,33 e 2,06 para mulheres, respectivamente.^21^

Embora o IMC elevado constitua um fator de risco independente para IC, múltiplas investigações têm mostrado uma associação reversa entre o IMC e a mortalidade, levando ao conceito do “paradoxo da obesidade.” Um dos primeiros estudos em 2001, com 1.203 pacientes com ICFEr avançada, mostrou que IMC > 27,8 kg/m^2^ estava associado a um benefício de sobrevida estatisticamente significativo.^5^ Uma análise da sobrevida hospitalar e IMC em mais de 100.000 pacientes com IC descompensada identificou uma redução de 10% no risco de mortalidade para cada aumento de 5 unidades no IMC.^22^ Adicionalmente uma meta-análise incluindo > 22.000 pacientes com IC crônica mostrou que o risco de mortalidade cardiovascular e hospitalização era menor em pacientes com sobrepeso (risco relativo de 0,79 e 0,92, em comparação com IMC normal, respectivamente).^23^ Na nossa coorte de pacientes com IC, os pacientes com maior IMC também apresentaram melhor prognóstico (Figura 1).

Historicamente, o VO_2_p tem sido a variável do teste de exercício cardiorrespiratório mais amplamente usada para determinar o prognóstico da IC e o momento de transplante.^11^ Porém, outras variáveis, incluindo a inclinação VE/VCO_2_, também são fortes preditores prognósticos.^19^ A vantagem adicional de medir a inclinação VE/VCO_2_ é que este valor continua confiável se um paciente não atingir o esforço máximo (RER > 1,05) e, portanto, não atingir seu “verdadeiro” VO_2_p. ^24^

No nosso estudo, a inclinação VE/VCO_2_ e o VO_2_p eram fortes preditores prognósticos. Chase et al.,^12^ demonstraram que a inclinação VE/VCO_2_ mantém o seu valor prognóstico independente do IMC em pacientes com IC.^12^ Também demonstramos que IMC elevado resulta em um resultado melhor na análise não ajustada. Porém, quando a inclinação VE/VCO_2_ ou o VO_2_p foram levados em consideração, o IMC perdeu a sua capacidade prognóstica. Além disso, quando os pacientes foram agrupados de acordo com a sua classe de aptidão cardiorrespiratória, o IMC não influenciou os desfechos. Analisando a nossa população com IC por classe de IMC, também pudemos observar que os pacientes com maior IMC apresentaram melhores parâmetros prognósticos (incluindo FEVE, níveis de sódio e inclinação VE/VCO_2_), indicando que estes pacientes apresentavam um quadro de IC menos avançado.

Estes achados indicam que o paradoxo da obesidade pode ser mitigado ou mesmo negado pela aptidão cardiorrespiratória, podendo representar apenas um viés de sobrevivência ou de evento-índice. A IC é um estado catabólico e o IMC elevado pode representar uma reserva metabólica, enquanto o IMC mais baixo pode ser uma consequência da perda de peso não intencional e da caquexia cardíaca, que está associada a um mau prognóstico.^25^ Adicionalmente, a experiência clínica das nossas Clínicas de Insuficiência Cardíaca tem demonstrado que os pacientes obesos podem apresentar maior comprometimento funcional devido ao aumento da massa corporal e, portanto, procurar atendimento médico mais cedo, o que leva à implementação mais precoce de terapia prognóstica. Além disso, é possível que alguns dos pacientes identificados como “obesos”, de fato, tenham aumento de massa muscular e força muscular.^26^

O paradoxo da obesidade já foi questionado em outros estudos. Lavie et al.,^16^ demonstraram que, em pacientes com ICFEr, o IMC foi um preditor de sobrevida no grupo com baixa aptidão cardiorrespiratória (VO_2_p < 14 mL/kg/m^2^), mas não no grupo com alta aptidão cardiorrespiratória.^16^ Mais recentemente, Piepoli et al.,^27^ verificaram que o papel prognóstico do IMC sumiu quando idade, sexo, FEVE e VO_2_p foram levados em consideração.^27^

Estes estudos anteriores que avaliaram a influência da aptidão cardiorrespiratória no paradoxo da obesidade analisaram apenas a influência do VO_2_p, que é dependente do esforço e altamente influenciado pela motivação do paciente.^28^ No nosso estudo, também demonstramos que a inclinação VE/VCO_2_, que é um parâmetro independente do esforço máximo, mitigou o paradoxo da obesidade. Portanto, a relação entre a aptidão cardiorrespiratória e o paradoxo da obesidade não é influenciada pelo esforço máximo do exercício realizado durante o teste.

Apesar do benefício da perda de peso na prevenção de remodelação cardíaca adversa, IC e outras doenças cardíacas, não há um consenso claro em relação à perda de peso em pacientes com IC. Grandes ensaios clínicos são necessários para compreender melhor os benefícios e os riscos da redução de peso em pacientes com IC. Dado o estado atual das evidências, pode ser razoável aconselhar a perda de peso proposital, particularmente nos pacientes com graus mais graves de obesidade, incorporando os benefícios da atividade física, do treinamento físico e da aptidão cardiorrespiratória.^29^^,^^30^

### Limitações

Este é um estudo unicêntrico, o que limita a generalização dos resultados. No entanto, isto possibilitou que o protocolo do teste de exercício cardiorrespiratório fosse homogêneo em todos os casos e pode ter reduzido o número de médicos responsáveis pela interpretação do exame, diminuindo a variabilidade interobservador. Além disso, trata-se de uma população de pacientes com ICFEr (FEVE sistólica média 27,4% ± 7,3%) que eram capazes de realizar exercícios e, portanto, os resultados podem não se aplicar a toda a população com IC. Outra limitação é que os pacientes com maior IMC apresentaram menor RER. No entanto, estes tiveram o maior desempenho de exercício e a análise com a inclinação VE/VCO_2_ supera essa limitação, considerando que é um parâmetro independente do esforço máximo.

## Conclusão

Na população com IC, o IMC não esteve relacionado aos desfechos quando as variáveis do teste de exercício cardiorrespiratório foram levadas em consideração. Portanto, a aptidão cardiorrespiratória afeta a relação entre o IMC e a sobrevida em pacientes com IC.
