# Supplementary material for: O Impacto da Aptidão Cardiorrespiratória no Paradoxo da Obesidade em Insuficiência Cardíaca com Fração de Ejeção Reduzida
Source: Arq Bras Cardiol. 2020 Oct 13;115(4):639–45. [Article in Portuguese] doi: 10.36660/abc.20190337 (PMC8386967; doi:10.36660/abc.20190337)
Supplement: Translation

# Translation: Impact of Cardiorespiratory Fitness on the Obesity Paradox in Heart Failure with Reduced Ejection Fraction

**Authors:** Rita Ilhão Moreira, Tiago Pereira Silva, António Valentim Gonçalves, Joana Feliciano, Pedro Rio, Rui Soares, Rui Cruz Ferreira

**Affiliations:** 1 Lisboa Portugal Hospital de Santa Marta, Lisboa – Portugal

**Keywords:** Heart Failure, Obesity, Body Mass Index, Breathing Exercises, Stroke Volume, Cardiorespiratory Fitness, Respiratory Function Tests

## Abstract

**Background:**

Higher body mass index (BMI) has been associated with improved outcomes in heart failure with reduced ejection fraction. This finding has led to the concept of the obesity paradox.

**Objective:**

To investigate the impact of exercise tolerance and cardiorespiratory capacity on the obesity paradox.

**Methods:**

Outpatients with symptomatic heart failure and left ventricular ejection fraction (LVEF) ≤ 40%, followed up in our center, prospectively underwent baseline comprehensive evaluation including clinical, laboratorial, electrocardiographic, echocardiographic, and cardiopulmonary exercise testing parameters. The study population was divided according to BMI (< 25, 25 – 29.9, and ≥ 30 kg/m^2^). All patients were followed for 60 months. The combined endpoint was defined as cardiac death, urgent heart transplantation, or need for mechanical circulatory support. P value < 0.05 was considered significant.

**Results:**

In the 282 enrolled patients (75% male, 54 ± 12 years, BMI 27 ± 4 kg/m^2^, LVEF 27% ± 7%), the composite endpoint occurred in 24.4% during follow-up. Patients with higher BMI were older, and they had higher LVEF and serum sodium levels, as well as lower ventilatory efficiency (VE/VCO_2_) slope. VE/VCO_2_ and peak oxygen consumption (pVO_2_) were strong predictors of prognosis (p < 0.001). In univariable Cox regression analysis, higher BMI was associated with better outcomes (HR 0.940, CI 0.886 – 0.998, p 0.042). However, after adjusting for either VE/VCO_2_ slope or pVO_2_, the protective role of BMI disappeared. Survival benefit of BMI was not evident when patients were grouped according to cardiorespiratory fitness class (VE/VCO_2_, cut-off value 35, and pVO_2_, cut-off value 14 mL/kg/min).

**Conclusion:**

These results suggest that cardiorespiratory fitness outweighs the relationship between BMI and survival in patients with heart failure.

## Introduction

Obesity impacts most cardiovascular disease risk factors, and it is an independent risk factor for the development of heart failure (HF), being present in approximately 20% to 30% of patients with advanced HF.^1^^–^^3^ However, multiple investigators have demonstrated that elevated body mass index (BMI) is paradoxically associated with improved clinical outcomes in the setting of established HF, which has been termed the “obesity paradox”.^4^^–^^6^

Various competing and often contradictory mechanisms have been proposed to explain the HF obesity paradox. Possible reasons include increased levels of serum lipoproteins,^7^ low levels of adiponectin,^8^ and decreased response to sympathetic activation.^9^ Confounding factors have also been suggested as a potential explanation.^10^

Cardiorespiratory fitness, measured variously as peak oxygen uptake (pVO_2_) or ventilatory efficiency slope (VE/VCO_2_ slope), has been identified as an important predictor of survival in HF.^11^^,^^12^ A strong obesity paradox has been demonstrated in patients with coronary heart disease,^13^^,^^14^ but not in patients with high levels of exercise tolerance.^15^^,^^16^

We aim to investigate the impact of exercise tolerance and cardiorespiratory capacity on the obesity paradox.

## Methods

The investigation conforms to the principles outlined in the Declaration of Helsinki. All participants filled out a written informed consent form, and the institutional ethics committee approved the study protocol.

### Selection of patients and complementary evaluation

We performed a prospective cohort study including all patients with HF with reduced ejection fraction (HFrEF) (≤ 40%), in New York Heart Association (NYHA) class II or III, followed in the Heart Failure Clinics of our institution. All patients referred to the Heart Failure Clinics underwent comprehensive complementary evaluation, from 2000 to 2009. Clinical, laboratorial, electrocardiographic, echocardiographic, and cardiopulmonary exercise test data were prospectively collected; all exams were performed within a period of one month for each patient.

Patients with any of the following factors were excluded: age under 18 years old, planned percutaneous coronary revascularization or cardiac surgery, exercise-limiting comorbidities (including cerebrovascular disease, musculoskeletal impairment, and severe peripheral vascular disease), and previous heart transplant.

A maximal symptom-limited treadmill cardiopulmonary exercise test was performed using the modified Bruce protocol (GE Marquette Series 2000 treadmill). Minute ventilation, oxygen uptake, and carbon dioxide production were obtained breath-by-breath, using a SensorMedics Vmax 229 gas analyzer. Before each test, the equipment was calibrated in standard fashion using reference gases. Patients were encouraged to perform exercise until the respiratory exchange ratio (ratio between carbon dioxide production and oxygen consumption, RER) was ≥ 1.10. The pVO_2_ was defined as the highest 30-second average achieved during exercise and was normalized for body mass; surrogate for fat-free mass was considered in obese patients (BMI ≥ 30 kg/m^2^). Percentage of predicted pVO_2_ was calculated according to Hansen et al^17^. The VE/VCO_2_ slope was calculated by least squares linear regression, using data acquired throughout the whole exercise^18^. The electrocardiographic data was interpreted by a physician during the exam. Weight and height were obtained, using a 110-CH Welmy anthropometric scale, before the cardiopulmonary exercise test was performed.

A GE Vivid 9 ultrasound system was used to acquire parasternal long- and short-axis views, as well as apical two-, three-, and four-chamber views. Echocardiographic parameters, including left ventricle end-diastolic and end-systolic volumes and left ventricle ejection fraction, were determined according to the American Society of Echocardiography's recommendations.

### Follow-up and endpoint

All patients were followed-up for 60 months. Patients were evaluated for the occurrence of death, heart transplant, or the need for mechanical circulatory support. Data was obtained from the outpatient clinic visits and review of medical charts, with a complementary standardized telephone interview for all patients at 12, 36, and 60 months of follow-up.

The combined endpoint was defined as cardiac death, urgent heart transplant (occurring during unplanned hospitalization due to worsening of HF, requiring inotropes), or need for mechanical circulatory support.

### Statistical analysis

Patients were divided into the following three groups according to BMI: < 25, 25 – 29.9, and ≥ 30 kg/m^2^. Cardiorespiratory fitness was dichotomized into low- and high-risk according to VE/VCO_2_ (cut-off value of 35^19^) and pVO_2_ (cut-off value of 14 mL/kg/min^11^). Categorical data are presented as frequencies (percentages), and continuous variables as mean (standard deviation), as appropriate. Continuous variables were analyzed using the unpaired Student's t test after normality was verified (Kolmogorov-Smirnov test); categorical variables were analyzed using the chi-squared or Fisher's exact tests. One-way analysis of variance (ANOVA) was used for between-group comparison, when appropriate. Univariable and multivariable Cox regression models were applied to analyze time until the combined endpoint. Survival was estimated by Kaplan-Meier analysis and compared by log-rank test. Further analysis of the lower BMI group (< 25 kg/m^2^) was performed, separating BMI < 20 and BMI 20 – 24.9 kg/m^2^. However, due to the small percentage of patients with BMI < 20 kg/m^2^ (only 17 patients), only baseline characteristics were evaluated (Supplementary Table S1 and Supplementary Figure S1), and no further statistical analysis was performed. All statistical tests were two-sided. P value < 0.05 was considered significant. SPSS version 21 software (SPSS Inc., Chicago, Illinois, USA) was used for computation.

## Results

A total of 282 patients were included, with mean age of 53.7 ± 12.1 years; 75.5% were male, with mean BMI 26.8 ± 4.3 kg/m^2^, and 37.6% had ischemic cardiomyopathy. Mean left ventricular ejection fraction (LVEF) was 27.4% ± 7.3%, and 23.0% of patients were in NYHA class ≥ III. Regarding therapy, 96.8% were receiving an angiotensin-converting enzyme inhibitor or angiotensin receptor blocker; 80.1% were receiving a beta-blocker; 68.1% were receiving a mineralocorticoid antagonist, and 26.2% had biventricular pacing. All patients were followed up during 60 months. The combined endpoint of cardiac death, urgent heart transplant, or need for mechanical circulatory support occurred in 24.4% of patients.

### Body mass index groups

Baseline characteristics of patients according to BMI groups are shown in Table 1. Patients with higher BMI were older, and they had higher LVEF and serum sodium levels. Exercise effort was, on average, maximal in all BMI groups (RER > 1.05), although higher BMI was associated with lower RER value. Elevated BMI was associated with lower VE/VCO_2_ slope (p 0.005), as well as numerically higher pVO_2_ and percentage of predicted pVO_2_, which did not reach statistical significance, however.

**Table 1:** Baseline characteristics according to BMI class

Baseline characteristics	BMI < 25 kg/m^2^ (n = 99)	BMI 25 – 29.9 kg/m^2^ (n = 119)	BMI ≥ 30 kg/m^2^ (n = 64)	p
Age, years, mean (SD)	49.0 (± 9.6)	59.3 (± 4.5)	57.3 (± 8.5)	0.022
Male sex, n (%)	70 (70.7%)	92 (77.3%)	51 (79.7%)	0.359
Ischemic etiology, n (%)	37 (37.4%)	44 (37.0%)	25 (39.1%)	0.961
Diabetes mellitus, n (%)	8 (8.0%)	30 (25.2%)	22 (35.1%)	<0.001
LVEF, % mean (SD)	24.0 (± 5.2)	28.3 (± 0.6)	27.8 (3.6)	0.003
NYHA class ≥ III, n (%)	29 (29.6%)	24 (20.3%)	12 (19.0%)	0.184
ACE inhibitors / ARBs, n (%)	98 (99.0%)	113 (95.0%)	62 (96.9%)	0.241
Beta-blockers, n (%)	75 (75.8%)	99 (83.2%)	53 (82.8%)	0.335
Mineralcorticoid antagonist, n (%)	63 (63.6%)	86 (72.3%)	44 (68.6%)	0.364
Biventricular pacing, n (%)	21 (21.2%)	33 (27.7%)	19 (29.7%)	0.402
ICD, n (%)	23 (23.2%)	30 (25.2%)	16 (35.0%	0.938
Hb, g/dL mean (SD)	15.0 (± 1.3)	12.4 (± 1.1)	13.6 (± 1.7)	0.075
eGFR, mL/min/1.73 m^2^ mean (SD)	103.4 (± 48.5)	69.0 (± 23.3)	73.0 (±23.5)	0.140
Sodium, mEq/L mean (SD)	134.5 (± 7.1)	139.0 (±2.6)	136.4 (± 4.8)	0.025
BNP, pg/mL mean (SD)	534.3 (± 365.3)	350.7 (± 89.0)	573.4 (± 300.6)	0.710
RER, mean (SD)	1.13 (± 0.14)	1.06 (± 0.49)	1.07 (± 0.15)	0.023
pVO_2_, mL/kg/min mean (SD)	15.0 (± 2.6)	15.2 (± 3.9)	16.1 (± 2.8)	0.758
% predicted pVO_2_, % mean (SD)	43.0 (± 8.4)	55.3 (± 9.3)	60.3 (± 16.1)	0.207
VE/VCO_2_ slope, mean (SD)	43.4 (± 6.6)	33.8 (± 6.0)	33.1 (± 8.1)	0.005

BMI: body mass index; LVEF: left ventricular ejection fraction; NYHA: New York Heart Association; ACE inhibitors: angiotensin-converting enzyme inhibitors; ARB: angiotensin receptor blocker; ICD: implantable cardioverter defibrillator; Hb: hemoglobin; eGFR: estimated glomerular filtration rate; BNP: brain natriuretic peptide; RER: respiratory exchange ratio; pVO_2:_ peak oxygen consumption; VE/VCO_2_ slope: ventilator efficiency slope. P calculated by analysis of variance.

In an unadjusted Cox proportional hazards model, BMI was a predictor of event-free survival when expressed as a continuous variable (hazard ratio [HR] 0.940, CI 0.886 – 0.998, p 0.042, Table 2) or a dichotomous variable (log-rank p value 0.047, Figure 1).

**Table 2:** Composite endpoint according to unadjusted body mass index and cardiopulmonary exercise test parameters

Dependent variable	HR (95% CI)	p
BMI, unadjusted	0.940 (0.886 – 0.998)	0.042
VE/VCO_2_ slope, unadjusted	1.164 (1.135 – 1.194)	< 0.001
pVO_2_, unadjusted	0.791 (0.742 – 0.842)	< 0.001

BMI: body mass index; HR: hazard ratio; VE/VCO_2_ slope: ventilator efficiency slope; pVO_2_: peak oxygen consumption.

**Figure 1:** Kaplan-Meier analysis according to body mass index (BMI) in the overall group.

### Cardiorespiratory fitness

Both VE/VCO_2_ slope and pVO_2_ were strong predictors of event-free survival in univariable analysis (p < 0.001, Table 2).

When patients were grouped into low- and high-risk cardiorespiratory fitness classes according to VE/VCO_2_ slope, BMI was not a predictor of clinical outcomes on univariate Cox regression analysis (p 0.771 for VE/VCO_2_ slope > 35 and p 0.439 for VE/VCO_2_ slope ≤ 35). Figure 2 illustrates the event-free survival characteristics of each cardiorespiratory fitness group. Furthermore, BMI did not affect event-free survival when patients were grouped for pVO_2_ (p 0.170 for pVO_2_ ≤ 14 mL/kg/min and p 0.164 for pVO_2_ > 14 mL/kg/min).

**Figure 2:** Kaplan-Meier analysis according to body mass index (BMI) in the (A) low cardiorespiratory fitness group (VE/VCO_2_ slope > 35) and (B) high cardiorespiratory fitness group (VE/VCO_2_ slope ≤ 35).

Although BMI was a predictor of prognosis on univariable analysis, after using a Cox regression analysis adjusting for VE/VCO_2_ slope, BMI lost its prognostic capacity (p 0.786, Table 3). Moreover, there was no relationship between BMI and event-free survival after adjusting for pVO_2_ (p 0.201, Table 3).

**Table 3:** Composite endpoint according to body mass index adjusted to cardiopulmonary exercise test parameters.

Dependent variable	HR (95% CI)	p
BMI, adjusted by VE/VCO_2_	1.008 (0.949 – 1.072)	0.786
BMI, adjusted by pVO_2_,	0.949 (0.892 – 1.020)	0.201

BMI: body mass index; HR: hazard ratio; VE/VCO_2_ slope: ventilator efficiency slope; pVO_2:_ peak oxygen consumption.

## Discussion

In this study, we evaluated whether cardiorespiratory capacity affects the obesity paradox. The findings can be summarized as follows: (1) obesity paradox is present in this HF population; (2) VE/VCO_2_ slope and pVO_2_ are strong prognostic predictors, and (3) most importantly, the prognostic capacity of BMI is lost when considering either of these two cardiorespiratory fitness parameters.

Obesity is a major risk factor for the development of HF. In the Framingham Heart Study, with 5,881 participants, the risk of HF doubled in obese subjects (HR 1.90 for men and HR 2.12 for women).^20^ These results were similar in larger studies, including one with over 59,000 participants free of HF at baseline, where the multivariable-adjusted HRs for developing HF at different levels of BMI (< 25, 25 – 29.9, and ≥ 30 kg/m^2^) were 1.00, 1.25, and 1.99 for men and 1.00, 1.33, and 2.06 for women, respectively.^21^

Although elevated BMI constitutes an independent risk factor for HF, multiple investigations have shown a reverse association between BMI and mortality, leading to the concept of “obesity paradox.” One of the first studies in 2001, with 1,203 patients with advanced HFrEF, showed that BMI > 27.8 kg/m^2^ was associated with a statistically significant survival benefit.^5^ An analysis of in-hospital survival and BMI in more than 100,000 patients with decompensated HF identified that mortality risk was lowered by 10% for every 5-unit increase in BMI.^22^ Furthermore, a meta-analysis including > 22,000 patients with chronic HF showed that the risk of cardiovascular mortality and hospitalization was lowest in overweight patients (relative risk 0.79 and 0.92 compared to normal BMI, respectively).^23^ In our cohort of patients with HF, patients with higher BMI also presented better prognosis (Figure 1).

Historically, pVO_2_ has been the cardiorespiratory exercise test variable most widely used for determining HF prognosis and timing of transplant.^11^ However, other variables, including VE/VCO_2_ slope, are also strong predictors of prognosis.^19^ The additional advantage of VE/VCO_2_ slope measurement is that its value is still reliable if a patient does not reach a maximal effort (RER > 1.05) and therefore does not achieve his or her “true” pVO_2._ ^24^

In our study, VE/VCO_2_ slope and pVO_2_ were both strong predictors of prognosis. Chase et al.^12^ demonstrated that VE/VCO_2_ slope maintains prognostic value irrespective of BMI in patients with HF^12^. We also demonstrated that higher BMI conveys a better outcome in unadjusted analysis. However, when either VE/VCO_2_ slope or pVO_2_ were considered, BMI lost its prognostic capacity. Moreover, when the patients were grouped according to their cardiorespiratory fitness class, BMI did not influence outcomes. Analyzing our HF population by BMI class, we were also able to observe that patients with higher BMI had better prognostic parameters (including LVEF, sodium levels, and VE/VCO_2_ slope), indicating that these patients presented a less advanced HF condition.

These findings indicate that the obesity paradox might be mitigated and even negated by cardiorespiratory fitness, and it may only represent a survival or index event bias. HF is a catabolic state, and elevated BMI may represent metabolic reserve, while lower BMI may be a consequence of unintentional weight loss and cardiac cachexia, which is associated with poor prognosis.^25^ Additionally, the clinical experience in our Heart Failure Clinics has shown that obese patients may experience greater functional impairment due to increased body mass and therefore seek medical assistance first, leading to earlier implementation of prognostic therapy. Additionally, it is possible that some of the patients identified as “obese,” in fact, have increased muscle mass and muscular strength.^26^

The obesity paradox has previously been challenged in other studies. Lavie et al.^16^ demonstrated that, in patients with HFrEF, BMI was a significant predictor of survival in the group with low cardiorespiratory fitness (pVO_2_ < 14 mL/kg/m^2^), but not in the high cardiorespiratory fitness group.^16^ More recently, Piepoli et al.^27^ verified that the prognostic role of BMI disappeared when age, gender, LVEF, and pVO_2_ were taken into consideration.^27^

These previous studies that evaluated the influence of cardiorespiratory fitness on the obesity paradox only analyzed the influence of pVO_2_, which is effort-dependent and highly influenced by patient motivation.^28^ In our study, we also demonstrated that VE/VCO_2_ slope, which is a maximal effort-independent parameter, mitigated the obesity paradox. Therefore, the relationship between cardiorespiratory fitness and the obesity paradox is not influenced by maximal exercise effort performed during the test.

Despite the benefit of weight loss in the prevention of adverse cardiac remodeling, HF, and other cardiac diseases, there is no clear consensus regarding weight loss in patients with HF. Large clinical trials are needed to better understand the benefits and risks of weight reduction in patients with HF. Given the current state of evidence, it may be reasonable to advise purposeful weight loss, particularly in those with more severe degrees of obesity, incorporating the benefits of physical activity, exercise training, and cardiorespiratory fitness.^29^^,^^30^

### Limitations

This is a single center study, which limits the generalization of results. Nevertheless, this made it possible for the cardiorespiratory exercise test protocol to be homogeneous in all cases, and it may have reduced the number of physicians responsible for interpretation of the exam, thus reducing interobserver variability. Additionally, the population comprised patients with HFrEF (mean systolic LVEF 27.4% ± 7.3%) who were able to perform exercise and, therefore, the results may not apply to the entire HF population. A further limitation is that patients with higher BMI presented lower RER. However, these patients had the highest exercise performance, and analysis with VE/VCO_2_ slope overcomes this limitation, as it is a maximal effort-independent parameter.

## Conclusion

In the studied HF population, BMI was not related to outcomes when cardiorespiratory exercise test variables were taken into consideration. Therefore, cardiorespiratory fitness affects the relationship between BMI and survival in HF patients.
